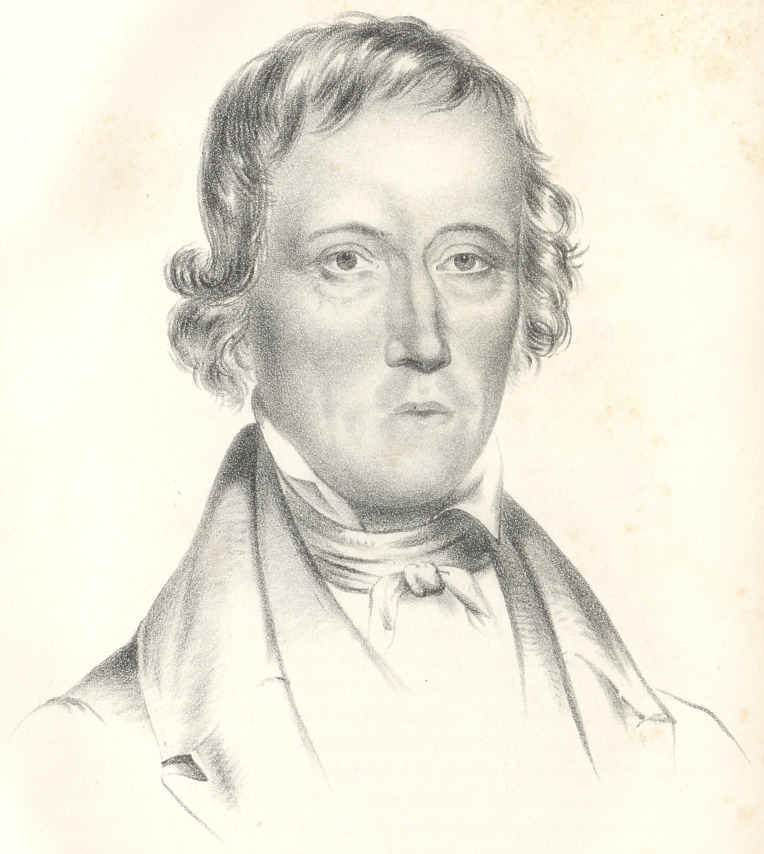# A Case of the Cheiloplastic Operation, with Two Portraits

**Published:** 1850-01

**Authors:** Abraham Stout

**Affiliations:** Easton, Pennsylvania


### MEDICAL EXAMINER,

AND

RECORD OF MEDICAL SCIENCE.

NEW SERIES.—NO. LXI.—JANUARY, 1850.

### ORIGINAL COMMUNICATIONS.

### *A Case of the Cheiloplastic Operation, with two portraits.* By ABRAHAM STOUT, M. D., of Easton, Pennsylvania.

In the month of November, 1844, David Steward, of Kingston township, Luzerne county, State of Pennsylvania, consulted me on account of an extensive disease of his lips. The report which he gave of himself was, that he was then fifty-three years of age, and a farmer by occupation; had always enjoyed good health, and lived a very regular and temperate life. In 1839 the disease began with a chap in the middle of the lower lip, from which an excrescence grew and enlarged gradually to the attainment of the present size; it involved the whole of the lower lip, extended around both the angles of the mouth, and occupied about one half of the upper lip.

The disease consisted of a tumour of a scirrho-cancerous nature, irregular on its surface, and extending nearly to the base of the inferior maxillary bone on the right side. [See the portrait No. 1, which gives a correct representation of the disease.] A yellowish sanies was constantly oozing from the whole surface of the tumour, which was about four times the thickness of a healthy lip; it was hard and not painful. It extended in the mouth to the gums, and loosened the two middle incisor teeth.

At the first examination of the disease, I pronounced it incurable. This greatly depressed the spirits of the patient, who remarked, that he had come a great distance with a determination to have this horrible disease removed, and he did not like to return home without having something done for him, however great his suffering might be. This remark induced me to take his case into more serious consideration. I examined the glands in the neck and face carefully, and found no disease in either. I then told him that an operation might possibly succeed, and made him acquainted with the nature and extent of the one required to give him the least chance of being cured. He immediately consented.

After his system had been duly prepared, I performed the operation in the following manner, in the presence of Doctors Cooper, Henry, Innis, Abernethy, Lachenaur and Swift. The patient being seated on a chair, and his head supported by an assistant, a thin piece of shingle was introduced between the lip and the teeth. I commenced an incision in the sound part of the upper lip, and carried my scalpel in a circle around the tumor into the cheek, opposite the left angle of the mouth, on the left side of his face, then obliquely downwards and forwards to a point nearly at the base of the inferior maxillary bone. From this point an arched incision was made in front of the chin to a corresponding point on right side, and from here upwards and backwards into the right cheek opposite the angle of the mouth, and from here a circular incision above the disease into the sound portion of the upper lip. Ligatures were now put upon the bleeding arteries, and the diseased mass removed by cutting it close from the inferior maxillary bone. At this stage of the operation, the patient felt faint, and we were obliged to give him wine and a few minutes rest. After he had revived, I extracted the four inferior incisor teeth, and then removed the gums and alveolar processes by sawing on each side of the space which the teeth had occupied, and in extent a little below the termination of the sockets of the teeth. The projecting alveoli were next removed with a chisel and the bone nippers. This constituted the second stage of the operation, when the patient again required some wine and rest. As soon as he had regained his strength, I cut the soft parts from the anterior surface of the jaw bone, and on each side as far back as the centre of the space between the middle of the chin and the angles of the bone, and down about midway from the base of the chin to the upper part of the pomum adami, continuing in progress anterior to the platysma myoides muscle. The flap was then drawn forwards and upwards, and the edge brought into contact with the edge of the remaining upper lip, and secured on each side by several twisted sutures, leaving space enough for a mouth. A compress of lint was laid over the chin, and supported by adhesive straps so as to keep the flap in close contact with the parts under it, in order to promote their union. Here terminated the third and last stage of the operation. A large dose of wine was now given to the patient, and he was put to bed. He soon recovered from the shock of the operation, and afterwards did remarkably well. He was nourished by giving him drink and Indian gruel frequently from the spout of a tea-pot. On the fourth day after the operation, the twisted sutures were removed, when the parts had completely united by the first intention. On the tenth day after the operation, the portrait No. 2 was taken by Mr. Samuel Moore, a very talented artist, and it is a faithful representation of the patient's face as it was then. His mouth was rather too small at first; but the lips have gradually stretched so much, that it is now, for all purposes, large enough. About a year after he had returned home, he accidentally bit his lower lip, from which cause it inflamed and became very sore; but the inflammation soon subsided, and the sore healed without any difficulty, and has continued well up to the present period, April 24th, 1848.

*Remarks.*—The soreness and inflammation in David Steward's lip, in consequence of his biting it, may be looked upon as a test establishing the important fact, that the disease for which he has been operated upon, is not likely to return. Had any predisposition of a return of the disease existed, it would, in all probability, have been called into action by the soreness and inflammation.

Before I had separated the flap for the formation of the artificial lip from its connection to the inferior maxilla, I apprehended the necessity of making a transverse cut into the flap under the jaw bone, to enable me to bring the edge, or artificial lip, in contact with the upper lip; but I was surprised at the great ease with which it was brought up without such a cut. I could not only readily bring it into contact with the upper lip, but with the nose also; and I would not be surprised, if, in the future, surgeons would form both upper and lower lips, and consequently a complete artificial mouth, from the integuments of the chin.

The case of David Steward strongly admonishes us not to come to a conclusion too hastily, and turn our patients off by pronouncing their cases incurable. Had he not insisted upon it that he would have something done for the removal of his disease before he would return home, he might perhaps to this day be laboring under that disgusting disease, and I might still consider his case incurable. I think now, that a surgeon ought to advance as far as any other surgeon has ever gone. and them venture a step further, for the comfort and prolongation of the life of his patient.

## Figures and Tables

**Figure f1:**
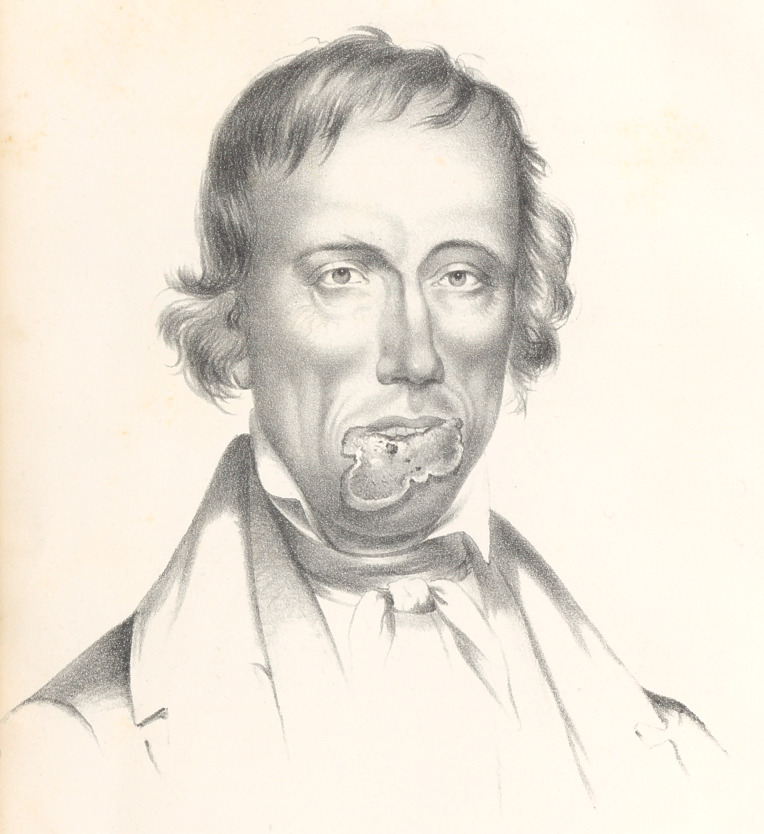


**Figure f2:**